# Errors in Funding/Support Statement and Conflict of Interest Disclosures

**DOI:** 10.1001/jamanetworkopen.2026.42535

**Published:** 2026-10-08

In the Original Investigation titled “Periprocedural Mortality Risk After Elective Atrial Fibrillation Ablation in Japan,”^1^ published August 29, 2026, there was an incorrect grant number in the Funding/Support statement and a typographical error in the Conflict of Interest Disclosures. This article has been corrected.^1^
